# Skin Grafting on 3D Bioprinted Cartilage Constructs In Vivo

**DOI:** 10.1097/GOX.0000000000001930

**Published:** 2018-09-14

**Authors:** Peter Apelgren, Matteo Amoroso, Karin Säljö, Anders Lindahl, Camilla Brantsing, Linnéa Stridh Orrhult, Paul Gatenholm, Lars Kölby

**Affiliations:** From the * Department of Plastic Surgery, University of Gothenburg, The Sahlgrenska Academy, Institute of Clinical Sciences, Sahlgrenska University Hospital, Göteborg, Sweden; † Department of Clinical Chemistry and Transfusion Medicine, Institute of Biomedicin, Sahlgrenska University Hospital, Göteborg, Sweden; ‡ Department of Chemistry and Chemical Engineering, 3D Bioprinting Centre, Chalmers University of Technology, Göteborg, Sweden.

## Abstract

**Background:**

Three-dimensional (3D) bioprinting of cartilage is a promising new technique. To produce, for example, an auricle with good shape, the printed cartilage needs to be covered with skin that can grow on the surface of the construct. Our primary question was to analyze if an integrated 3D bioprinted cartilage structure is a tissue that can serve as a bed for a full-thickness skin graft.

**Methods:**

3D bioprinted constructs (10 × 10 × 1.2 mm) were printed using nanofibrillated cellulose/alginate bioink mixed with mesenchymal stem cells and adult chondrocytes and implanted subcutaneously in 21 nude mice.

**Results:**

After 45 days, a full-thickness skin allograft was transplanted onto the constructs and the grafted construct again enclosed subcutaneously. Group 1 was sacrificed on day 60, whereas group 2, instead, had their skin-bearing construct uncovered on day 60 and were sacrificed on day 75 and the explants were analyzed morphologically. The skin transplants integrated well with the 3D bioprinted constructs. A tight connection between the fibrous, vascularized capsule surrounding the 3D bioprinted constructs and the skin graft were observed. The skin grafts survived the uncovering and exposure to the environment.

**Conclusions:**

A 3D bioprinted cartilage that has been allowed to integrate in vivo is a sufficient base for a full-thickness skin graft. This finding accentuates the clinical potential of 3D bioprinting for reconstructive purposes.

## INTRODUCTION

The restoration of the wing of the nose and auricle requires 2 fundamentally different biological entities, a cartilaginous framework and skin coverage. Functionally, the air flow is compromised through a collapsed nostril and the hearing is impaired if the auricle is absent. Cosmetically, absence of an ear or a damaged nostril represents a stigmatizing appearance, and reconstruction of these structures today often includes a step-by-step autologous transplantation of cartilage from conchae to the nose or the costochondral joint for reconstruction of the auricle.^1–6^ These procedures are often multistaged and also afflicted by significant donor-site morbidity.^1,7^ Furthermore, the restoration of a 3-dimensional, complex structure requires specific surgical and artistic skills, but the reconstruction is still often less than perfect.^1,8^

Other reconstructive approaches to recreate an auricular scaffold have previously been evaluated. One example of an approved and readily available biomaterial is polyethylene (Medpor, Stryker, Minneapolis, Minn.).^9,10^ These implants have some beneficial features compared with standard rib cartilage grafts such as, significantly better grades of ear definition and size match and reduced surgical time and morbidity.^11,12^ However, other studies point out the risk for implant extrusion and the risk for soft-tissue necrosis complications due to the need for coverage with a temporoparietal fascial flap, which is often required in an implant procedure.^13–15^

From a reconstructive point of view, cartilage constitute a tissue with several advantages. Cartilage is tolerant to hypoxia, and auto transplanted cartilage therefore has a good ability to integrate with the surrounding tissue without shrinkage or development of necrotic deformations.^16,17^ This is an absolute prerequisite for a good long-term result of the reconstruction. The capacity to tolerate hypoxia makes chondrocytes a rewarding cell type for regenerative medicine. Utilizing the 3D bioprinting technology, cartilage can also be shaped in detail into sturdy 3-dimensional structures, such as a framework for an auricle. The harvesting, shaping, and the time consuming, multistaged surgical procedures can thereby, in part, be omitted.

The 3D bioprinting technology offers a new approach where cartilaginous structures can be regenerated with autologous cells dispersed in a biocompatible supporting framework, that is, bioink. The 3D shape of the bioprinted construct can be very precise and invasive harvesting procedures, for example, from rib cartilage, is not necessary.^18,19^ In previous studies, chondrocyte proliferation and formation of cartilage occurs in the 3D bioprinted scaffolds.^20,21^ Furthermore, these studies confirm the chondrogenic boosting effect of stem cells, which reduce the amount of cartilaginous tissue needed, and the suitability of the nanocellulose/alginate framework.

Both reconstruction of the nose wing and the auricle require that the underlying cartilage structure is covered with skin. One of the principal problems with auricle reconstruction is the local sparsity of skin. Often, the final result is an auricle with a surface much more flattened than desired. Therefore, covering the bioprinted cartilage framework with a skin graft that could attach tightly to the cartilage surface and also bring out and accentuate the delicate shape could be a way forward. A 3D bioprinted cartilage framework for an auricle has the potential to be very elaborate in shape, but would still lack skin coverage that allows these high-resolution shapes to be emphasized. The aim of the present study was therefore to evaluate if an integrated 3D bioprinted cartilage construct has the capacity to serve as a bed for a full-thickness skin graft.

## MATERIALS AND METHODS

### Cells and Skin

Human bone marrow–derived mesenchymal stem cells (hBM-MSC) originated from a female donor (Rooster Bio, Frederick, Md.). Human nasal chondrocytes (hNC) were harvested from a male donor undergoing nasoseptal reconstruction at the Department of Otorhinolaryngology of Ulm University Medical Centre (Ulm, Germany). The harvesting was approved by the Ethical Advisory Board at Ulm University, Ulm, Germany (Dnr 152/08). The hBM-MSCs were cultured using an hBM-MSC High Performance Media Kit (RoosterBio, Frederick, Md.) in 37°C, humidified air with addition of 5% CO_2_, passaged after 4 days and harvested for printing on day 8. The hNC were cultured in Dulbecco’s modified Eagle medium/F12 (LifeTechnologies, Waltham, Mass.) supplemented with 10% fetal bovine serum (HyClone; GE Healthcare, South Logan, Utah) and 1% penicillin/streptomycin (HyClone; GE Healthcare) for 6 days before printing.

The full-thickness skin graft used for all 21 mice was harvested from a euthanized mouse.

### Bioinks and 3D Bioprinting

The bioink and the cells were mixed with a cell mixer (CELLINK AB, Gothenburg, Sweden) at a 11:1 ratio. The final cell density was 10 M cells/mL. A 10 × 10 × 1.2 mm grid was printed using nanofibrillated cellulose/alginate bioink (NFC/Alg) (CELLINK AB) with 0.6 mm spacing in an extrusion 3D bioprinter (INKREDIBLE; CELLINK AB). After printing, constructs were cross-linked with 100 mM CaCl_2_ for 5 minutes in 37°C. The constructs were washed with Hank´s balanced salt solution (HyClone; GE Healthcare). The printed constructs were then immediately implanted subcutaneously into the mice.

### Animals

Twenty-two, 8-week old, female, nude mice Balb/C (Scanbur, Karlslunde, Denmark) were used applying national and local directions. The study was approved by the Ethical Committee for animal experiments at Sahlgrenska University Hospital/Gothenburg University, Göteborg, Sweden (Dnr 36–2016).

### Experimental Design

**Fig. 1.**
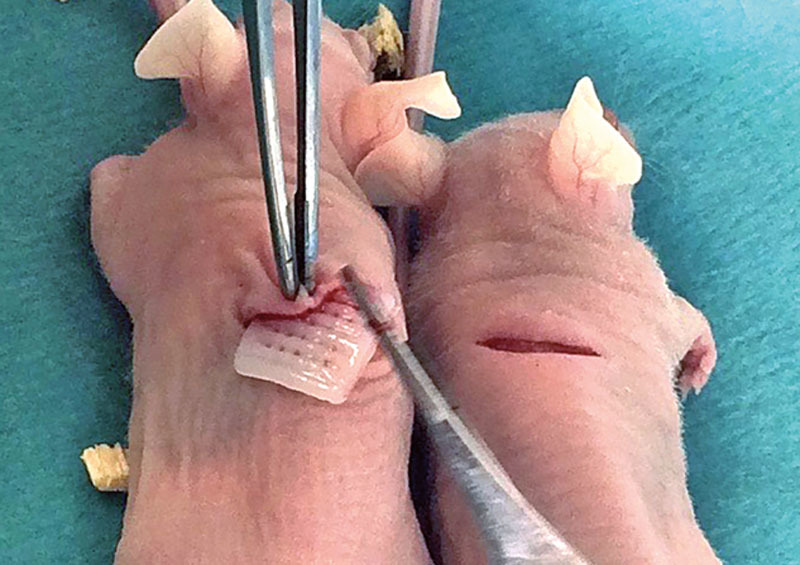
Subcutaneous implantation of the 3D bioprinted cell-laden constructs in 8-week-old naked mice.

The animals were put under general anesthesia induced by intraperitoneal injection of a mixture of Ketamine (50 mg/ml) and Medetomidine (1 mg/ml) in a 1:1 ratio. Each animal received 0.04 ml anesthetic solution per 20 g body weight. One 3D bioprinted construct was surgically implanted in a subcutaneous pocket on the back of each mouse (Fig. 1). All constructs were identical in composition. The skin pockets were then closed with Vicryl Rapid (Ethicon, Sommerville, N.J.).

Each animal carried the 3D bioprinted constructs for 45 days and were then, once again, put under general anesthesia. The back-skin pockets were opened and a 10 × 10 mm full-thickness skin graft from the donor mouse was transplanted on to the 3D printed constructs and fixated with Prolene 8-0 sutures. The pocket was again closed with Vicryl, and sealed with wound tape. After 60 days, half of the mice were randomly chosen and euthanized, and the constructs were explanted. The other group was instead put under general anesthesia a third time. The pocket roof was removed, and the surrounding skin was sutured edge-to-edge to the transplanted skin covering the 3D printed construct. The area was then covered with wound tape.

After 75 days, the constructs were harvested and fixated in 4% buffered formaldehyde supplemented with 20 mM CaCl_2_ overnight at 4°C and embedded in paraffin (Table 1).

**Table 1.**
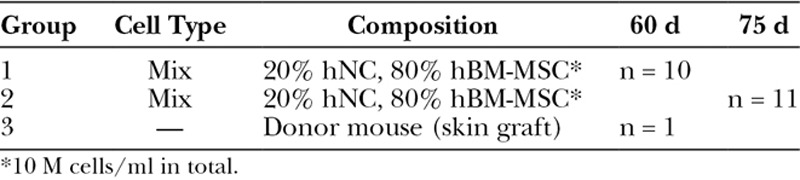
Experimental Design, Composition of the 3D Constructs

### Morphological Analysis

After deparaffinization, the sections were stained with Alcian Blue and van Gieson and scanned using a Nikon Eclipse 90i epi-fluorescence microscope equipped with a Nikon DS-Fi2 colour head camera and NIS-Elements imaging software suite (Version D4.10.02; Nikon Instruments Inc, Melville, N.Y.). Each section was morphologically analyzed with special attention to signs of dehiscence, disintegration, or necrosis.

### Ethical Approval

The study was approved by the Ethical Committee for animal experiments at Sahlgrenska University Hospital/Gothenburg University, Göteborg, Sweden (Dnr 36–2016).

## RESULTS

### Animals

One mouse in group 1 died of unknown cause on day 33 after the initial implantation. The others thrived during the trial period, and no signs of infection were registered.

### Cell-laden Bioinks

The cell-laden NFC/Alg bioinks could be printed with high printing fidelity and good printability and dimension stability. The explanted constructs had retained their integrity and structural properties macroscopically.

### Skin

**Fig. 2.**
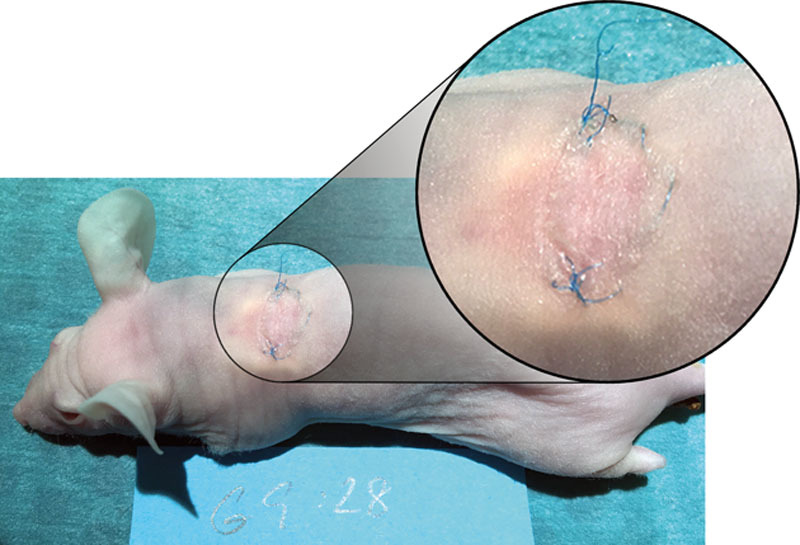
Complete integration of the full-thickness skin graft on top of the bioprinted cartilaginous construct 75 days after implantation and 30 days after the full-thickness skin graft transplantation.

The transplanted skin survived subcutaneously in all the 20 surviving animals. No necrosis was observed. The exteriorized skin in group 2 also survived and engrafted without any macroscopic signs of complications (Fig. 2). However, some transplants were partly covered by crusts and debris from the healing process.

**Fig. 3.**
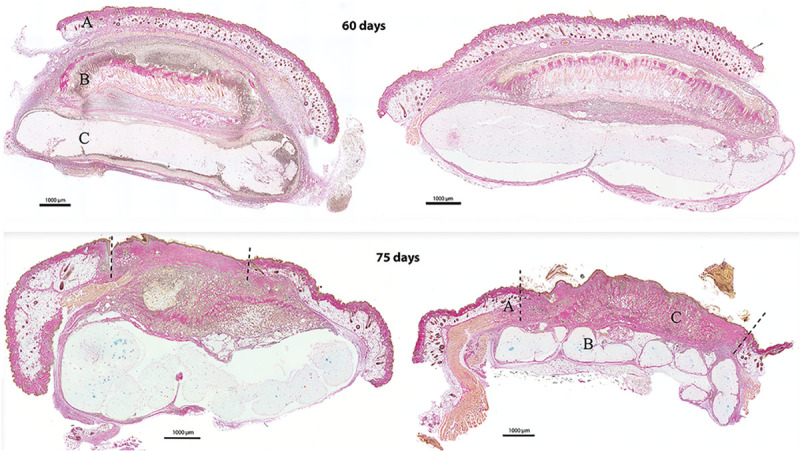
Histologic sections after Alcian Blue and van Gieson staining. Group 1 after 60 days (top) and group 2 after another 15 days (in total 75 days) with the graft exposed (bottom). A, The cutaneous pocket with a roof of native skin. B, Full-thickness skin graft. C, 3D bioprinted construct. Dotted lines denote the border between native skin and the transplant. All scale bars represent 1,000 µm.

**Fig. 4.**
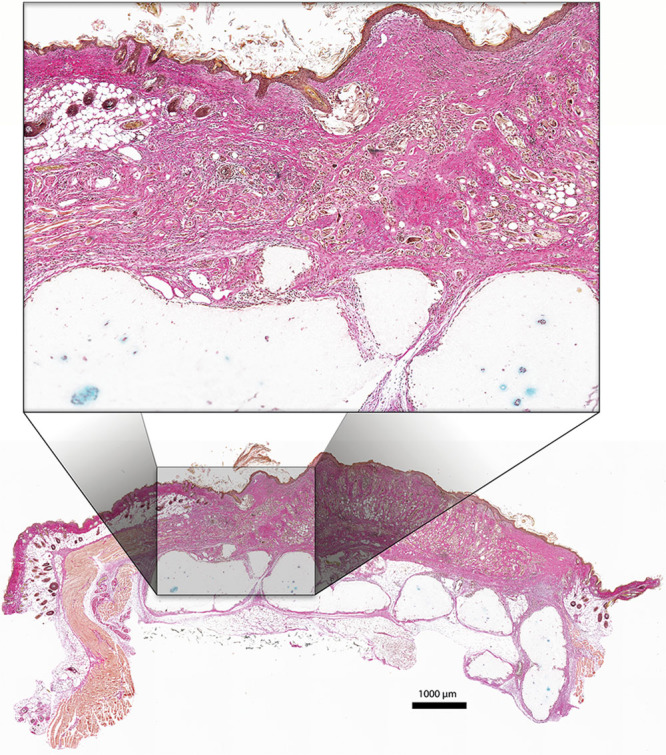
Histologic section after staining with Alcian Blue and van Gieson. The exposed full-thickness skin graft (group 2) is well integrated to the native skin bordering the wound area and also to the underlying cartilage construct. No signs of necrosis or dehiscence. Scale bar = 1,000 µm.

Microscopically, the transplants incorporated well in the histological layers with no signs of necrosis or detachment (Figs. 3, 4). However, lymphocytic infiltration was observed as a sign of inflammatory activity.

## DISCUSSION

The present study was designed to create a 3D bioprinting set-up similar to the clinical situation, where a patient is in need of a reconstruction of a composite structure, such as an auricle. We could affirm that the skin transplant attached to and grew on the bioprinted cartilage construct and that the quality of the grafted skin was good and withstood exposure to the environment. A clinically conceivable auricular reconstructive procedure with the 3D bioprinted cellulose scaffolds could start with an outpatient appointment. Imaging of the contralateral auricle, harvesting of autologous cells, mixing with bioink, printing, and then bioincubation of the construct on the forearm or subcutaneously on the abdomen, are carried out during the initial visit. Several weeks later, the patient is scheduled for surgery where the construct and the auxiliary vessels (eg, radial artery and vein) are harvested. The construct is attached on site by microsurgical anastomosis to temporal vessels. Lastly, a full, or split skin graft is transplanted on top of the construct.

The bioprinted cartilage constructs and, most importantly, the surrounding fibrous capsule obviously was able to support the skin graft structurally, and with nutrients and oxygen delivery. The diffusion limit for oxygen and nutrients is ~ 100–200 µm, and constructs larger than this requires vascularity.^22^ Hence, the size of the 3D bioprinted construct are a limiting factor. In the present study, the majority of the chondrocytes did not survive, maybe due to nutrition and oxygen deficiency. Kang et al.^18^ demonstrated in 2016 a method to overcome the diffusion limit utilizing micro channels inside the constructs, thereby making it possible to print larger constructs. Also, further studies on immune-specific species are needed to corroborate this setup under the influence of an efficient immune response.

Additionally, since rodents heal by withering, they may not be the ideal model for studying the human healing process in all aspects,^23–26^ our purpose was instead to analyze a basic question; if the vascularization of the fibrous capsule surrounding the 3D bioprinted construct is sufficient to support the skin transplant. Moreover, rodents’ skin lacks apocrine sweat glands and is proportionally much thinner than in humans and also comprises an additional muscle layer that makes the comparability difficult.^27,28^

Furthermore, the short study time is a limitation of the present study. The long-term outcome of shape stability, elastic features, and tissue integrity are crucial factors that have to be addressed in further studies.

## CONCLUSIONS

A 3D bioprinted cartilage that has been allowed to integrate in vivo is a sufficient base for a full-thickness skin graft. This finding accentuates the clinical potential of 3D bioprinting for reconstructive purposes.

## ACKNOWLEDGMENTS

The authors acknowledge Thomas Möller and Dr. Hector Martinez for assistance with 3D-bioprinting experiments. The authors also acknowledge Professor Nicole Rotter at the Department of Otorhinolaryngology, University Medical Centre, Ulm, Germany, for providing the human nasal chondrocytes.
